# High mobility group box 1 protein (HMGB1) as biomarker in hypoxia-induced persistent pulmonary hypertension of the newborn: a clinical and *in vivo* pilot study

**DOI:** 10.7150/ijms.34344

**Published:** 2019-08-06

**Authors:** Zhen Tang, Min Jiang, Zhicui Ou-yang, Hailan Wu, Shixiao Dong, Mingyan Hei

**Affiliations:** 1 Department of Pediatrics, the Third Xiangya Hospital of Central South University, Changsha, Hunan, 410013 China; 2 Neonatal Center, Beijing Children's Hospital, Capital Medical University, Beijing, 100045 China

**Keywords:** Hypoxia, Newborn, infants, Persistent pulmonary hypertension of the newborn (PPHN), High mobility group box-l (HMGB1), Rat

## Abstract

**Background:** Inflammation plays an important role in neonatal hypoxia-induced organ damage. Newborns with perinatal asphyxia often develop persistent pulmonary hypertension of the newborn (PPHN). The objective of this study was to explore changes in the pro-inflammatory high mobility group box-l (HMGB1) protein during hypoxia-induced PPHN clinically and *in vivo*.

**Methods:** Serum samples were collected from full-term newborns at PPHN onset and remission. As controls, blood serum samples were collected from the umbilical arteries of healthy full-term newborns born in our hospital during the same period. Clinical data for neonates were collected and serum levels of HMGB1, IL-6, and TNF-α were detected by enzyme-linked immunosorbent assay (ELISA). An animal study compared a PPHN Sprague-Dawley rat model to healthy newborn control rats. Histopathology was used to evaluate changes in the pulmonary artery wall. ELISA and western blot analyses were used to examine HMGB1 levels in the serum and lungs.

**Results:** Serum HMGB1 levels were significantly elevated in newborns with PPHN, compared to those in healthy controls, and decreased dramatically after PPHN resolution. HMGB1 changes were positively correlated with serum tumor necrosis factor-α (TNF-α) and interleukin-6 (IL-6) levels. Histopathological analysis demonstrated that the median wall thickness of pulmonary arterioles accounting for the percentage of pulmonary arteriole diameter (MT%) was not significantly different between PPHN and control groups 3 d after PPHN, although thickness of the small pulmonary arterial wall middle membrane and stenosis of the small pulmonary arteries. ELISA and western blot analyses showed similar trends between serum HMGB1 levels and HMGB1 protein expression in the lungs. Serum and lung HMGB1 levels were significantly elevated soon after PPHN onset, peaked after 24 h, and then decreased after 3 d, although they remained elevated compared to those in the control group.

**Conclusions:** This study indicates that HMGB1 is related to hypoxia-induced PPHN pathogenesis. HMGB1 changes might thus be used as an early indicator to diagnose hypoxia-induced PPHN and evaluate its improvement. We also provide important evidence for the involvement of inflammation in the progression of hypoxia-induced PPHN.

## Introduction

In addition to hypoxic-ischemic brain damage, persistent pulmonary hypertension of the newborn (PPHN) is not uncommon in newborns with perinatal asphyxia. PPHN is a syndrome characterized by the sustained elevation of pulmonary vascular resistance, which results in poor lung perfusion and further aggravates systematic and brain tissue hypoxia, resulting in deterioration of the patient's condition and increased mortality and morbidity 1, 2. As a systemic syndrome, PPHN has a variety of causes, but lacks an early and effective diagnostic method 3, 4. PPHN diagnosis is currently performed by determining the pulmonary artery pressure based on cardiac catheterization and echocardiography 5, 6, which are not practical for newborns. Inflammation plays an important role in neonatal hypoxia-induced organ damage. One clinical study indicated that serum levels of a variety of inflammatory factors are significantly increased during adult pulmonary arterial hypertension (idiopathic pulmonary arterial hypertension, IPAH) mediated by chronic hypoxia, including tumor necrosis factor-alpha (TNF-α) and interleukin-6 (IL-6). Further, IPAH prognosis is closely linked to increases in these inflammatory factors 7, 8, illustrating the important role inflammation plays in the occurrence and development of IPAH. Recently, high-mobility group box-1 (HMGB1), an inflammatory mediator, has received increasing attention for its role in the pathogenesis of pulmonary arterial hypertension. Clinical studies confirmed that high levels of HMGB1 could be detected around the pulmonary artery walls of plexiform lesions in patients with IPAH, and that serum and alveolar lavage fluid HMGB1 levels in patients with IPAH were significantly increased 9. Animal experiments have also reported significant increases in serum HMGB1 levels in mouse models of chronic hypoxia, and demonstrated that exogenous recombinant HMGB1 can exacerbate pulmonary arterial hypertension, whereas the administration of HMGB1-neutralizing antibodies can slow its progression 10. Elevated serum levels of HMGB1 in a rat model of pulmonary arterial hypertension were found to mainly result from alveolar macrophages and smooth muscle cells 11. However, the effects of HMGB1 on neonatal PPHN remain unknown.

To determine whether similar changes in HMGB1 levels occur in PPHN, we first examined HMGB1 levels in the serum of full-term newborns admitted for perinatal hypoxia that rapidly progressed to PPHN. HMGB1 changes were also investigated using an animal model of PPHN to understand the role of HMGB1 in neonatal hypoxia-induced PPHN pathogenesis.

## Materials and Methods

### Research design

The study included both clinical studies and animal experiments. Clinical studies included a case group comprising newborns with no evidence of infection, who were admitted to the hospital with perinatal asphyxia and diagnosed with PPHN shortly thereafter, and a control group consisting of newborns born in the same hospital during the same period. With the parents' informed consent, umbilical cord blood samples and laboratory residual blood samples were collected from patients in the case group. After centrifugation, 100 μL of serum was collected and stored at -80 °C for further testing. Regarding ethical considerations, only healthy full-term neonatal cord blood samples were taken for the control group, following the same process as that performed for the case group. The animals were randomly divided into PPHN and normal control groups. At each designated time point, newborn rats were sacrificed by decapitation and 200 μL blood samples were taken. After centrifugation, 30 μL serum samples were collected and stored at -80°C. At the same time, the lungs of the animals were collected, and proteins were extracted and stored at -80 °C.

### Clinical research objectives

### Inclusion criteria for newborns with PPHN

The clinical component of this study was approved by the medical ethics committee of the Third Xiangya Hospital of Central South University and Beijing Children's Hospital, Capital Medical University. PPHN newborns with complete clinical data and an initial diagnosis of perinatal asphyxia who were diagnosed with PPHN within 3 d of birth were enrolled at the neonatal intensive care unit of the Third Xiangya Hospital of Central South University and Beijing Children's Hospital, Capital Medical University from January 2016 to December 2017. Formal written consent was signed by the parents of each infant enrolled in this study. PPHN diagnosis included perinatal history, clinical manifestations, and laboratory examinations, and was confirmed by color Doppler echocardiography 12. When the patient's oxygen index was < 20 and Doppler echocardiography showed a pulmonary artery systolic pressure ≤ 2/3 the systolic pressure, they were considered to be in PPHN remission 13, 14.

### Exclusion criteria for newborns with PPHN

Newborns were excluded based on any of the following criteria: (1) their mothers were diagnosed with chronic hepatitis B, acute and chronic pancreatitis, diabetes, autoimmune diseases, thyroid dysfunction, or cancer; (2) there was a serious infection during pregnancy, including sepsis or significant prenatal infection; (3) their mothers had a special medication history such as taking non-steroidal anti-inflammatory drugs or selective serotonin re-uptake inhibitors during pregnancy; (4) the newborns suffered from severe congenital cardiovascular disease, diaphragmatic hernia, severe pathological jaundice, or abnormal thyroid function. Newborns who died within 72 h or did not achieve remission allowing the cessation of treatment were excluded. According to the inclusion and exclusion criteria, 12 children with PPHN were included in the study.

### Inclusion criteria for the control group

Ten full-term newborns born at the Obstetrics department of the Third Xiangya Hospital of Central South University were included as a control group. The inclusion criteria were as follows: (1) a normal obstetric history for the mother, with no special medications during pregnancy (such as aspirin and other non-steroidal anti-inflammatory drugs); (2) a gestational age between 37-42 weeks, with the onset of feeding within 2 h after birth; (3) no cyanosis, shortness of breath, hypoxemia, or other abnormalities after birth; (4) postpartum care in the same ward as the mother without special medical intervention, and discharge from the hospital after 3-4 d.

### PPHN animal model using neonatal rats

Experimental animals were provided by the Hunan Agricultural University Experimental Animal Center. Three-day-old Sprague-Dawley rats were randomly divided into the control group (n = 40) and the hypoxia-induced PPHN group (n = 40). The animal study component of this study was approved by the medical ethics committee of the Third Xiangya Hospital of Central South University. The control group was housed without any intervention, while PPHN was induced in the PHHN group starting 4 d after birth, as previously described 15-17. Briefly, experimental animals were placed in a hypoxia chamber filled with 10% oxygen + 90% nitrogen for 7 consecutive days. The chamber temperature was maintained at 26 ± 0.5 °C, and anhydrous calcium chloride was placed in the chamber to absorb the carbon dioxide exhaled by the experimental animals. Chambers were opened briefly (< 15 min) for cleaning and other administration. The time of completion of the last 24 h hypoxia exposure was counted as 0 h (at 11 d of age) and the experimental animals were sacrificed at 2 h (11 d of age; n = 10), 8 h (11 d of age; n = 10), 24 h (12 d of age; n = 10), and day 3 (14 d of age; n = 10). Before the animals were sacrificed, four animals were randomly selected at the corresponding time points to measure mean pulmonary artery pressure (mPAP) according to previous literature 18. Briefly, pups were anesthetized by pentobarbital administration (50 mg/kg) via intraperitoneal injection, fixed and intubated, and connected to mechanical ventilation (HX-200 small animal ventilator, Taimeng Technology Co., Ltd., Chengdu, China). Setting respiratory rate was 100 breaths /min, tidal volume 0.2 ml, 5 minutes later, pups were opened the chest, exposed the right ventricle and inserted the catheter (OD: 0.5 mm) into the pulmonary artery root, connected to the other end of the catheter to the RM-6280 multi-channel intelligent physiological signal transducer (Chengdu SiChuan) to record mPAP. After the pulmonary artery pressure was measured, the same numbers of rats in the control group were sacrificed at the corresponding time points.

### Serum HMGB1 testing

Blood samples were respectively collected from newborns when they were diagnosed with PPHN and when PPHN was alleviated. Normal control blood samples were collected from the umbilical arterial blood. All blood samples from rats were collected when they were sacrificed by decapitation. Enzyme-linked immunosorbent assays were used to detect HMGB1 (IBM, Germany), TNF-α (Wuhan Huamei Biotech, China), and IL-6 (Wuhan Huamei Biotech, China), according to the manufacturers' instructions.

### Pulmonary vascular morphology

Four rats in each group were selected to detect vascular pathological changes on day 3. After decapitation, the right middle lobe tissue was removed and fixed in 4% paraformaldehyde for 24 h. After paraffin embedding, sections were sliced to 5 μm and stained with hematoxylin and eosin (H&E). After drying, morphological changes in pulmonary arterioles were examined under a light microscope. For each sample, three visual fields at 400× magnification containing intact transverse pulmonary arteries were randomly selected. MIAS-2000 medical image analysis software was used to calculate the median wall thickness of pulmonary arterioles accounting for the percentage of pulmonary arteriole diameter (MT%).

### Western blot analysis

The lungs of experimental animals were removed, washed with phosphate-buffered saline, trypsin digested, washed again, and lysed in RIPA lysis buffer (Shanghai Yesen Biotech, China); all operations were performed on ice. The samples were centrifuged at 13000 × *g* for 30 min and the supernatant was placed in a new tube. The protein content was determined by the Lowry method 19. Samples were aliquoted at 30 μg/tube and stored at -80 °C. After boiling for 5 min in sample buffer, protein samples were separated by sodium dodecyl sulfate-polyacrylamide gel electrophoresis at 110 V and 40 mA for 60 min. Proteins were transferred to PVDF membranes, blocked in 5% skimmed milk powder solution for 1 h, and incubated overnight at 4 °C with a mouse anti-HMGB1 monoclonal antibody (ab11354, Abcam, 1:1000). β-actin was used as a control. The membranes were washed with tris-buffered saline containing 0.5% tween 20 and then incubated for 1 h at 26 °C with a goat anti-mouse IgG horseradish peroxidase-labeled secondary antibody (HFA007, R&D, 1:5000). Bands were visualized based on enhanced chemiluminescence and exposure to film. Bands were analyzed using Quantity One TM 4.2.2 (Bio-Rad) software, and HMGB1 levels were expressed as the gray ratio of the target and control bands.

### Statistical analysis

SPSS 18.0 software was used for statistical analysis. Data with a non-normal distribution were expressed as the median (Q1-Q3). Normally distributed data were expressed as the mean ± standard deviation. The means of two groups were compared with two-independent sample t-tests. Comparisons of multiple groups were performed by one-way ANOVA followed by a least significant difference t (LSD-t) test for multiple comparisons. Comparisons of count data were performed using the Fisher exact method. Indicator correlations were examined by linear correlation analysis. P < 0.05 was considered statistically significant.

## Results

### Clinical results

#### General characteristics of the newborns in the two groups

There were no differences in gestational age, birth weight, mode of delivery, sex, or maternal age between the control and PPHN groups (all P > 0.05). However, there were significance differences in 1-min Apgar scores, as well as pH and HCO_3_^-^ concentrations in the umbilical cord blood (P < 0.05; Table 1). Two newborns with amniotic fluid contamination were reported in the PPHN group, and all membranes ruptured within 18 h before delivery. The newborns in the PPHN group were admitted at an average age of 1.00 h (0.63-9.00 h) and diagnosed with PPHN by bedside cardiac ultrasound 12.00 h (3.14-15.67 h) after admission. Blood gas analysis showed that the mean PaO_2_ value when the newborns were diagnosed with PPHN was 35.63 ± 8.30 mmHg, whereas the mean SaO_2_ value was 58.70 ± 13.30%. The difference in percutaneous oxygen saturation before and after catheterization was 10.07 ± 5.19% and the systolic pressure/systolic pressure ratio of the pulmonary artery was 0.56 ± 0.05 at the same time. The average time to PPHN remission was 75.6 h (52.1-96.5h), and newborns were hospitalized for an average of 14.9 d (11.4-17.7d).

#### Serum levels of HMGB1, TNF-α, and IL-6

**Figure 1:**
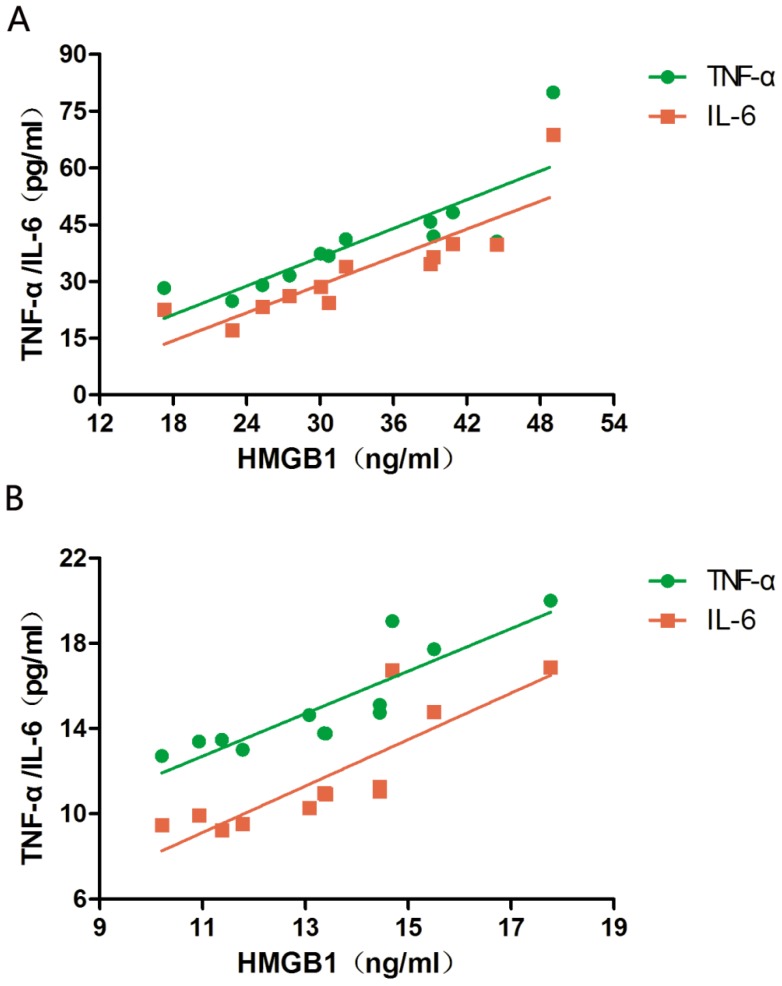
Correlation between serum HMGB1 levels and serum TNF-α/IL-6 levels in newborns with persistent pulmonary hypertension of the newborn (PPHN). a. Correlation between TNF-α/IL-6 levels and HMGB1 levels at PPHN onset. b. Correlation between TNF-α/IL-6 levels and HMGB1 levels after PPHN remission; n = 12.

The serum levels of HMGB1, TNF-α, and IL-6 at PPHN onset and at PPHN alleviation were 33.19 ± 9.45 vs. 13.42 ± 2.14 ng/mL, 40.41 ± 14.3 vs. 15.12 ± 2.45 pg/mL, and 32.98 ± 13.42 vs. 11.75 ± 2.77 pg/mL, respectively, and all differences were statistically significant (P < 0.05; Table 2). The serum levels of TNF-α and IL-6 were positively correlated with HMGB1 levels both at PPHN onset (r = 0.832 and 0.866, respectively, P < 0.05) and after remission (r = 0.873 and 0.843, respectively, P < 0.05; Figure 1).

### Animal experiment results

#### mPAP at each time point

**Figure 2:**
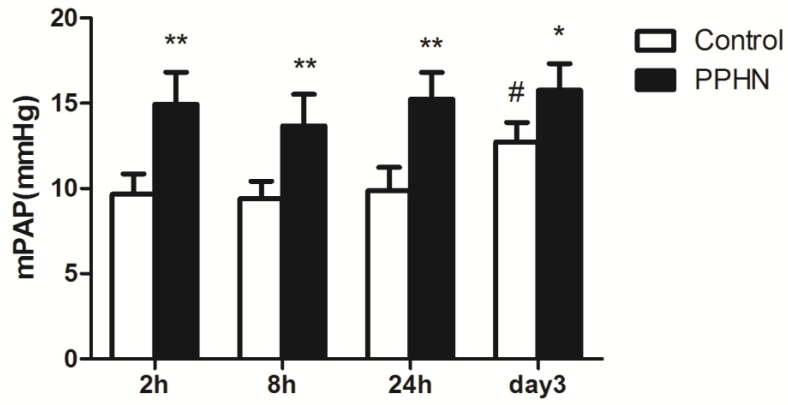
Changes in mean pulmonary artery pressure (mPAP) at different time points after hypoxia-induced persistent pulmonary hypertension of the newborn (PPHN). *P < 0.05 vs. control; **P < 0.01 versus control; #P < 0.05 vs. 24 h time point; n = 4.

The mPAP of the PPHN group was higher than that of control group at each time point (respectively, P < 0.05; Figure 2). Compared to that at the 24 h time point, mPAP was significantly increased on day 3 time point in the control group (P < 0.05; Figure 2), but was not increased in the PPHN group (P > 0.05; Figure 2).

#### Serum HMGB1 levels at each time point

**Figure 3:**
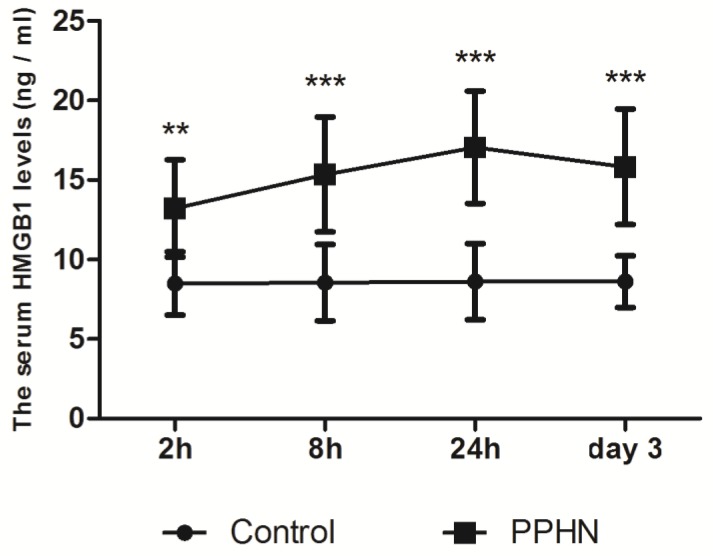
Trends in serum HMGB1 levels at different time points of persistent pulmonary hypertension of the newborn (PPHN) based on a rat model. Serum HMGB1 levels were stable at different time points in the control group; however, serum HMGB1 levels in the PPHN group first increased and then decreased. **P < 0.01 vs. control; ***P < 0.001 vs. control; n = 10.

Serum levels of HMGB1 were basically stable in 11-14 day old control rats. Within 0-3 days after PPHN, HMGB1 levels in the PPHN group increased by 1.5-2 fold compared to those in the control group at each time point, peaking 24 h after PPHN. Differences between the PPHN and control groups were statistically significant (respectively, P < 0.05; Figure 3), but there were no differences among different time points in the PPHN group (F = 2.134, P > 0.05).

#### Pulmonary vascular histopathology

**Figure 4:**
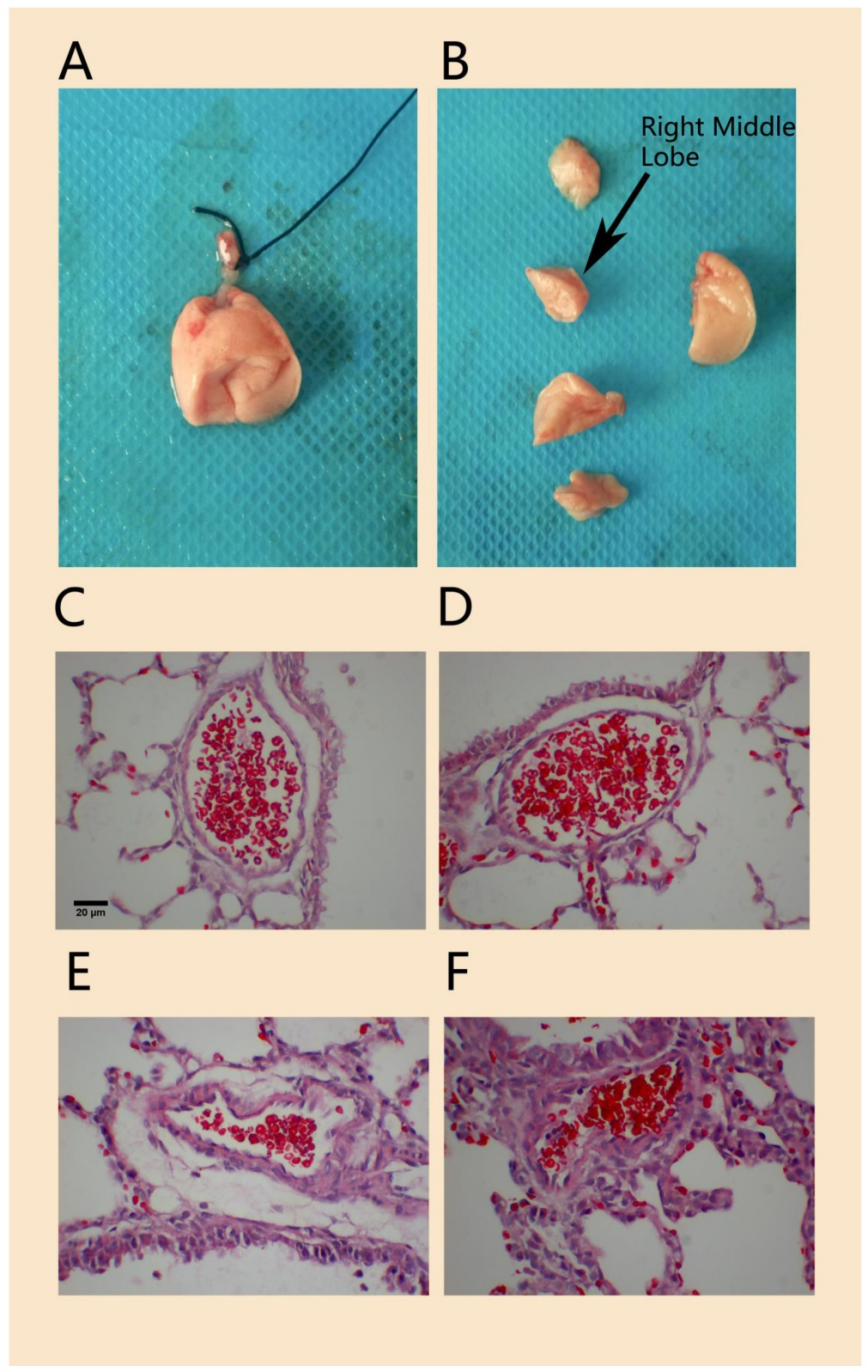
H&E staining of pulmonary arterioles in a rat model of PPHN. Morphology of lung tissue and right middle lobe in experimental neonatal rats**(A, B)**.The wall of pulmonary arterioles accompanied with terminal bronchioles in normal control rats **(C, D)** and in PPHN rats **(E, F)**. scale bar: 20 µm; n=4.

Compared to that in the normal control group, H&E staining of lung tissue in the hypoxia-induced PPHN animal model on day 3 showed that the wall of the pulmonary arteriole accompanied by the terminal bronchioles was thicker and that the lumen was narrower; the thickened arteriole wall was mainly caused by thickened medial vessel walls (Figure 4). The MT% in the PPHN group was 7.2 ± 4.9%, compared to 5.9 ± 4.7% in the control group. Although there was no significant difference between the two groups (P > 0.05), the MT% of the PPHN group increased by 20.6% compared to that in the control group.

#### Western blot analysis of lung tissue from PPHN rats

**Figure 5:**
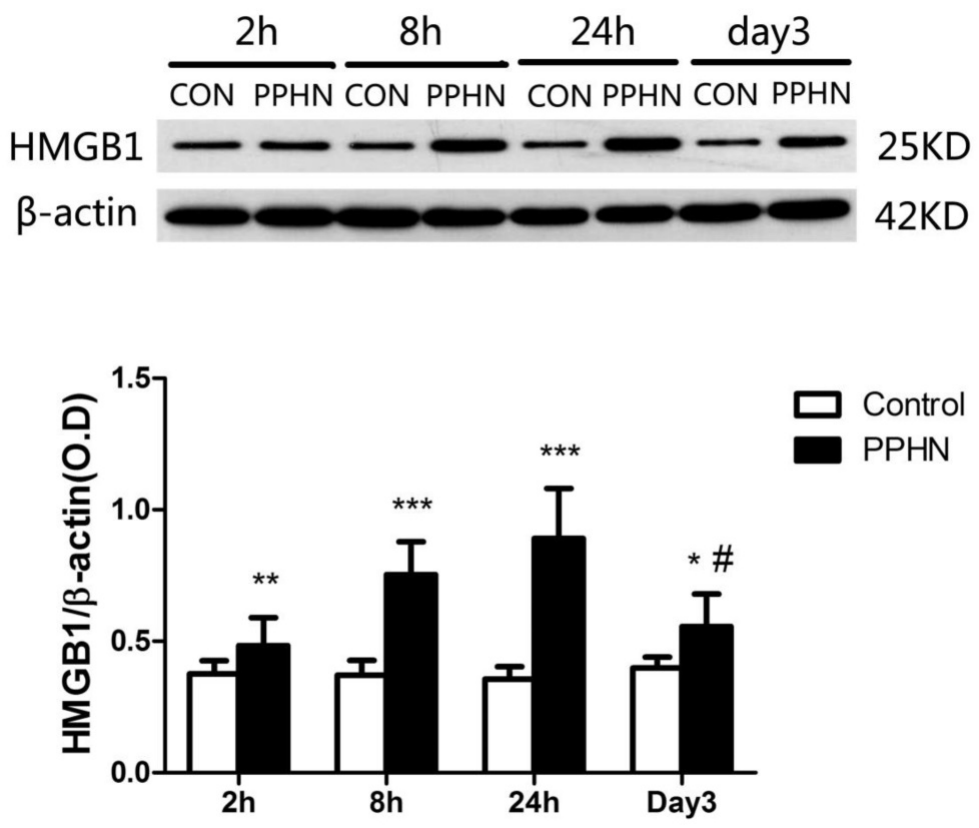
HMGB1 expression in the lungs of a rat model of PPHN as detected by western blotting. ***P < 0.001 vs. control; **P <0.01 vs. control; *P < 0.05 vs. control; #P < 0.001 vs. 24 h time point; n = 6-10.

HMGB1 expression in the lungs of PPHN neonatal rats was significantly higher than that in the control group at 2, 8, and 24 h, as well as day 3(respectively, P < 0.05; Figure 5). In the PPHN group, HMGB1 levels were significant different among different time points (F = 14.136, P = 0.000), and the expression trend showed a significant increase at 2 h, peaking at 24 h, and then a decrease at day3. HMGB1 levels at day 3 were significantly lower than those at 24 h in the PPHN group (P < 0.001; Figure 5).

## Discussion

PPHN comprises three types (primary, congenital, and secondary), with different mechanisms of pathogenesis. PPHN can also be divided into pulmonary vascular underdevelopment, pulmonary vascular mal-development, and pulmonary vascular maladaptation depending on different pathological changes. Clearly, the etiology and pathogenesis of PPHN are complex 20. In recent years, increasing numbers of studies have found that extensive inflammatory cytokine dysregulation can trigger and aggravate the abnormal contraction and proliferation of pulmonary vascular smooth muscle cells, resulting in pulmonary artery wall remodeling. This suggests that the abnormal expression of inflammatory cytokines is related to the occurrence and development of pulmonary hypertension, and accordingly, the role of inflammation in the development of pulmonary hypertension is being increasingly studied 21-23. This study found the following results: (1) serum levels of HMGB1 in newborns with PPHN were significantly increased early after PPHN onset, and then decreased after remission, and were positively correlated with levels of the classic inflammatory factors TNF-α and IL-6. (2) In a rat model of PPHN, 7-day continuous hypoxia can induce an increase in pulmonary artery pressure lasting for 3 d, and histopathological analysis also showed thickening of the medial vessels of pulmonary arterioles and lumenal stenosis, although pulmonary arteriole changes did not reach the level of irreversible vascular remodeling on the 3rd day after PPHN. (3) ELISA and western blot analyses demonstrated similar short-term increases in HMGB1 levels in the serum and lung tissue of PPHN rats after PPHN onset, peaking after 8-24 h, and slightly decreasing, but remaining significantly higher than those in the control group, at day 3. These results indicate that changes in HMGB1 levels are related to the occurrence and development of PPHN. In hypoxia-induced PPHN models, pulmonary arterial changes might be dominated by vasospasms, and during this mode of remodeling, HMGB1 levels are more sensitive to changes than tissue histology. This provides an objective basis for using HMGB1 as an early diagnostic marker of PPHN and to monitor the PPHN process.

HMGB1 is a single-chain polypeptide consisting of 215 amino acids. In disease states, this protein can promote local and systemic inflammatory responses through its passive secretion from necrotic cells and active secretion from immune cells including macrophages, dendritic cells, and natural killer cells. It can also regulate the immune reaction by promoting the production of other inflammatory cytokines and activating different immune cells 24, 25. HMGB1 is a central component of the inflammatory network, as its secretion has an amplifying effect and it can also regulate the secretion of other inflammatory cytokines 26. This clinical study demonstrated that serum HMGB1, TNF-α, and IL-6 levels are significantly increased with PPHN onset and significantly decreased with PPHN alleviation. TNF-α and IL-6 expression was significantly positively correlated with HMGB1 expression in the serum of newborns with PPHN. These results suggested that hypoxia-induced HMGB1 and inflammatory release are closely related to the occurrence and development of PPHN. There are two possible mechanisms that could explain these changes (as follows): (1) hypoxia-induced HMGB1 release from activated pulmonary macrophages causes inflammation and directly leads to vascular endothelial cell injury, endothelial cell gap widening, increases in endothelial permeability, and changes in pulmonary vascular endothelial cell cytoskeletal remodeling, proliferation, and contraction in a dose-dependent manner 27, 28. (2) Extracellular HMGB1 stimulates the production of reactive oxygen species and downstream inflammatory cytokines inducing oxidative stress and TNF-α, among others; meanwhile, with increases in pulmonary artery pressure, blood flow shearing force is increased and hypoxia is exacerbated. This would result in an increase in the generation of reactive oxygen species by vascular endothelial cells, vascular smooth muscle cells, and adventitial fibroblasts, further stimulating the expression of HMGB1 and its receptors, and forming a positive feedback loop 11. Animal results showed that both the serum concentrations of HMGB1 and the expression of HMGB1 in lung tissues began to increase 2 h after PPHN onset, peaked after 24 h, and decreased at day 3. This dynamic change over time is consistent with a previous report showing HMGB1 secretion into the peripheral blood 24-48 h after PPHN onset 29.

In this study, the average gestational age of newborns with PPHN was 39 weeks and the average birth weight was 3.3 kg, a suitable weight for full-term newborns. Most newborns had intrauterine hypoxia and were diagnosed with PPHN shortly after birth. Their peripheral blood HMGB1 and inflammatory cytokine levels were significantly increased, indicating a link between PPHN and the hypoxia-induced inflammatory response. The progression to pulmonary hypertension was associated with hypoxic pulmonary vasospasm and pulmonary vascular remodeling, which both manifested as pulmonary arteriolar stenosis. Pulmonary vascular remodeling was markedly increased with MT% as the main manifestation. When the MT% increases to a certain extent, PPHN changes are considered irreversible 30. Animal studies have reported that pulmonary arterial pressure in neonatal rats is significantly increased 3-5 d after hypoxia exposure, but increases in MT% were not obvious 31. We also observed thickening of the pulmonary arteriole walls, accompanied by pulmonary terminal bronchioles, and that the MT% was not different, as compared to that in the control group, 3 days after PPHN induction despite an increase in MT% of 20.6%. These results indicate that the main pathological change in the early stage of PPHN is vasospasm; however, it is possible that pulmonary vessels undergo vascular remodeling at this time, although it is unclear when irreversible remodeling of the pulmonary arteries occurs during hypoxia progression. At present, studies examining hypoxia-induced PPHN have mainly focused on mediators that lead to irreversible vascular remodeling, such as endothelin-1 18, thyroid hormone receptor interactor 6, cyclin D1 15, and vascular endothelial growth factor 32. Although the inhibition of HMGB1 reduces the right ventricular systolic pressure and pulmonary vascular remodeling in an adult rat model of hypoxia-induced pulmonary arterial hypertension (PAH) by blocking the interaction between HMGB1 and the TLR4 adaptor MD2, little is known about the effects of HMGB1-mediated inflammation on pulmonary vasculature in developing animals 33. Further studies are needed to explore the role of HMGB1 in irreversible hypoxia-mediated pulmonary artery remodeling and related mechanisms during PPHN. In addition, it is also important to further address the clinical value of HMGB1 as a diagnostic and predictive marker of adverse PPHN prognosis.

In summary, the clinical research presented in this study indicates that serum levels of HMGB1 in newborns with PPHN are significantly increased early after PPHN onset, and then decreased after remission, and that they are positively correlated with levels of inflammatory factors. Animal experiments confirmed that HMGB1 levels in the peripheral blood and lung tissue change with hypoxia-induced PPHN progression, although there was no significant pulmonary vascular remodeling in PPHN rats. As an inflammatory mediator, HMGB1 plays an important role in the early stages of hypoxia-induced PPHN, suggesting that it might be a useful early marker for PPHN diagnosis.

## Figures and Tables

**Table 1:** Comparison of clinical data in two groups

	Control group (n = 10)	PPHN group (n = 12)	*t /χ ²*	*P*
gestational age(w)	39.1 ± 1.1	39.0 ± 1.2	-0.181	0.859
birth weight (g)	3399.0 ± 410.5	3289.1 ± 556.3	-0.517	0.611
Male / female (n)	6/4	7/5	-	0.639
Cesarean section / peace (n)	3/7	5/7	-	0.454
mother's age (y)	31.9± 3.4	32.3± 3.7	0.279	0.783
1 minute Apgar score ≤7 (n)	0	11	-	0.000
Cord blood PH	7.25± 0.10	7.07 ± 0.06	-5.687	0.000
cord Blood HCO3^—^ (mol/L)	-6.7± 2.3	-13.6± 3.2	-5.763	0.000

**Table 2:** Serum HMGB1, TNF-α and IL-6 levels among three situations

Group	number	HMGB1 (ng/ml)	TNF-α (pg/ml)	IL-6 (pg/ml)
Control group	10	2.37 ± 0.88	3.14 ± 1.30	3.47 ± 0.90
PPHN onset	12	33.19 ± 9.45^a^	40.41 ± 14.3^a^	32.98 ± 13.42^a^
PPHN remission	12	13.42 ± 2.14^bc^	15.12 ± 2.45^bc^	11.75 ± 2.77^bc^
*F*		81.171	53.354	39.089
*P*		0.000	0.000	0.000

a: The PPHN onset group compared with the normal cord blood group: Pa._HMGB1_ = 0.000, Pa._TNF-α_ = 0.000, Pa._IL-6_ = 0.000; b: The after PPHN remission group compared with the normal cord blood group: Pb._HMGB1_ = 0.000, Pb._TNF-α_ = 0.003, Pb._IL-6_ = 0.024; c: The after PPHN remission group compared with the PPHN onset group:Pc._HMGB1_ = 0.000, Pc._TNF-α_ = 0.000, Pc._IL-6_ = 0.000.
